# Epigenetic Regulation of *αA-crystallin* in High Myopia-Induced Dark Nuclear Cataract

**DOI:** 10.1371/journal.pone.0081900

**Published:** 2013-12-03

**Authors:** Xiang-Jia Zhu, Peng Zhou, Ke-Ke Zhang, Jin Yang, Yi Luo, Yi Lu

**Affiliations:** Department of Ophthalmology, Eye and Ear, Nose, and Throat Hospital, Fudan University, Shanghai, China; Casey Eye Institute, United States of America

## Abstract

**Purpose:**

To assess the etiology of early-onset dark nucleus in high-myopic patients and its relationship with the epigenetic regulation of *αA-crystallin* (CRYAA).

**Methods:**

We reviewed clinical data from patients who underwent cataract surgery at our center in 2012. Lens epithelial samples were collected during capsulorhexis, whereas young lens epithelium was donated. Cataract type and severity were graded according to the Lens Opacity Classification System III (LOCS III). DNA methylation was analyzed by pyrosequencing the CpG islands of the *CRYAA* promoter in the following groups: Age-Related Cataract (ARC) Nuclear Color (NC) 2–3; High-Myopic Cataract (HMC) NC2–3; ARC NC5–6; HMC NC5–6; and in young lenses graded NC1. We analyzed CRYAA expression by real-time polymerase chain reaction (PCR), reverse transcription PCR, and immunohistochemistry.

**Results:**

The odds ratio of dark nucleus in high-myopic patients was 5.16 (95% confidence interval: 3.98–6.69; p<0.001). CpG islands in lens epithelial *CRYAA* promoter in the HMC NC5–6 Group exhibited the highest methylation of all the groups, but no statistically significant differences were evident between the HMC NC2–3 and ARC NC2–3 Groups. Likewise, CRYAA mRNA and protein levels in the HMC NC5–6 Group were significantly lower than the ARC NC5–6 Group and high-myopic controls.

**Conclusions:**

High myopia is a risk factor for dark nucleus. Downregulation of *CRYAA* via the hypermethylation of CpG islands in its promoter could underlie the earlier onset of dark nucleus in high-myopic patients.

## Introduction

High myopia, defined as myopia exceeding −6.00 diopters or axis length ≥ 26 mm, is a disorder that affects almost the entire human eye, from the anterior pole to the posterior pole. High myopia can be associated with high refractive errors, cataract, open-angled glaucoma, and retinopathy, all of which are recognized as significant problems of subspecialties related to ophthalmology [1], and more difficulties are encountered during surgery in such cases.

As the incidence of high myopia is much higher in Asia than elsewhere [2], it is likely that high-myopic cataract (HMC) is more common in Asia [3]. Several population-based studies [3]–[6] have examined the association between high myopia and cataract. For example, data from the Blue Mountains Eye Study [4] suggested that high myopia is associated with both nuclear and posterior subcapsular cataract. In another series of studies [2], [7], it was reported that nuclear cataract is strongly associated with high-axial myopia. Praveen *et al*. [7] also reported that cataract density is higher in patients with high myopia than in other groups of patients. However, high myopia is not associated with posterior subcapsular or cortical cataract.

We have noticed that some patients with high myopia develop dark nuclear cataract at an earlier age than high-myopic patients without a dark nucleus or patients with age-related cataract (ARC). Patients with high-myopic dark nuclear cataract show rapid cataract progression at a relatively young age, they usually have a brown or dark nucleus, and may have a longer axis. As the largest cataract surgery center in Eastern China, we perform cataract surgery on more than 2,000 patients with high myopia each year, and those with high-myopic dark nuclear cataract are referred to our center much earlier than other patients because of a rapid deterioration in their vision. However, cataract surgery is very hazardous in such patients because of the high risk of posterior capsule tear, vitreous prolapse [8], persistent postoperative corneal edema, and retinal detachment. Therefore, it is particularly important to determine the etiology of this disorder.

Alpha-crystallin, composed of two subunits, αA- and αB-crystallin, is the most abundant structural protein in the human lens [9], [10]. The biologic role of α-crystallin is to bind to lens proteins that denature over time, thereby maintaining long-term lens transparency. This is particularly important in the lens, as there is no protein turnover in the inner part [11]–[13] and, once synthesized, these proteins persist for an individual’s lifespan. Therefore, adequate expression of α-crystallin is vital for maintaining the transparency of the human lens. However, as we recently reported [14], αA-crystallin expression is significantly decreased in ARC compared with healthy controls as a result of hypermethylation of CpG islands in the promoter of *CRYAA*, the gene encoding αA-crystallin. Recent studies [15]–[17] have focused on the epigenetic regulation of gene expression, which alters DNA–transcription factor affinity through two mechanisms: DNA methylation and histone modification. In DNA methylation, addition of a methyl group to cytosine in DNA changes the electrostatic nature of chromatin. Hypermethylation of CpG islands in a promoter causes heterochromatin formation and gene silencing [16].

Very few studies have addressed the reasons why patients with high myopia show earlier-onset and higher-density cataract. We hypothesized that hypermethylation of CpG islands in the *CRYAA* promoter underlies the etiology of HMC as well. To test our hypothesis, we examined the differences in characteristics among high-myopic dark nuclear cataract, HMC without a dark nucleus, and age-related nuclear cataract. Cataract type and severity were graded according to the Lens Opacity Classification System III (LOCS III). DNA methylation was analyzed by pyrosequencing the CpG islands in *CRYAA* promoter. *CRYAA* expression was compared among high-myopic eyes with dark nuclear cataract, high-myopic eyes with Nuclear Color (NC) Grade 2–3 cataract, and eyes of normal axial length with nuclear cataract of similar severity.

## Methods

The Ethics Committee of the Eye and ENT Hospital, Fudan University, Shanghai, China, approved our collection and use of anterior capsule membranes from patients undergoing cataract surgeries. All samples were collected after obtaining written informed consent from patients, and the consent procedure was also approved by the ethics committee. All procedures adhered to the *Declaration of Helsinki* for research involving human subjects.

### Clinical data review

We identified patients with/without dark nuclear cataract (NC5–6/NC2–4) among those with ARC and HMC using our follow-up tables for 2012. We retrieved clinical data for these patients and calculated the prevalence of cataract of LOCS III Grade NC5–6 in different age groups of patients with ARC and HMC.

### Collection of lens anterior capsule membrane samples

Before surgery, routine ophthalmic examinations were carried out. Cataract type and severity were graded using the modified LOCS III. NC status was graded based on six slit lamp images. Three ophthalmologists independently and simultaneously graded each cataract.

Consecutive patients with nuclear cataract and a long (≥ 26 mm) or normal (21–23.99 mm) axis admitted to our clinic between September 2012 and January 2013 were considered for this study. Patients with glaucoma, uveitis, previous trauma, or other ocular diseases, and those with diabetes or malnutrition, were excluded from this study. Patients were then subdivided into four groups based on LOCS III grade: HMC NC2–3 (*n*  =  7); HMC NC5–6 (*n*  =  11); ARC NC2–3 (*n*  =  7); and ARC NC5–6 (*n*  =  8). The average LOCS III grades for these groups were 2.57, 5.45, 2.57, and 5.50, respectively.

Lens anterior capsule membrane samples were obtained by continuous curvilinear capsulorhexis during cataract surgery; only samples without blood or iris tissue contamination or damage to other intraocular structures were used. We also collected lens anterior capsule membrane samples from nine individuals aged 30–40 years classified as NC1 without cataract. These samples were obtained from eyes of normal axial length donated to the Eye Bank of the Eye and ENT Hospital, and were used as normal controls. The interval between death and the collection of cadaveric eyes was < 4 hours. Anterior capsule membranes were carefully collected from the center of lenses. We then compared groups defined by axial length and LOCS III scores.

### Pyrosequencing

Pyrosequencing was performed as previously described [14], [18]. The polymerase chain reaction (PCR) was performed with 0.2 M of each primer for biotin (forward: TGGGGATATGTAGTTATTTTGATAGGAG; reverse: CTAAACCCCCAACCCCATAACCAT) following bisulfite conversion. PCR products were bound to Streptavidin Sepharose® High Performance (Amersham Biosciences, Uppsala, Sweden). Sepharose® beads containing the immobilized PCR products were purified, washed, and denatured with 0.2 M NaOH, then rewashed with a Pyrosequencing Vacuum Prep Tool (Qiagen, Venlo, Limburg, The Netherlands). Next, 0.5 µM of the pyrosequencing primer (CAACCCCATAACCATC) was annealed to the purified single-stranded PCR products, and the resulting products (10 μl) were sequenced using the Pyrosequencing PSQ 96 HS System (Qiagen). For each locus, methylation status was analyzed individually as a T/C single nucleotide polymorphism using Pyro-QCpG software (Qiagen).

### Reverse transcription polymerase chain reaction and relative quantitative real-time polymerase chain reaction

As previously described [14], [19], *CRYAA* mRNA levels were assayed as follows. For reverse transcription polymerase chain reaction (RT-PCR), total RNA was extracted with TRIzol® Reagent (Life Technologies Corporation, Carlsbad, CA, USA). Quantification and purity of total RNA was measured by A260/A280 absorption. A total of 1 µg RNA was reverse-transcribed using the First Strand cDNA Synthesis Kit. The primers used in RT-PCR were as follows: *CRYAA*, forward: 5'-CCTGCTGCCCTTCCTGTCGT-3'; *CRYAA*, reverse: 5'-TCCTGGCGCTCGTTGTGCT-3'; *β-actin*, forward: 5′-AAGTACTCCGTGTGGATCGG-3′; and *β-actin*, reverse: 5′-TCAAGTTGGGGGACAAAAAG-3′. Real-time PCR reactions were performed with SYBR® Green PCR Master Mix (Roche Applied Sciences, Basel, Switzerland). Dissociation melting curve analysis and 1% agarose gel electrophoresis were used to check the specificity of the PCR amplification products. Quantification analysis of *CRYAA* mRNA was normalized against the housekeeping gene *β-actin* as an internal control. Relative multiples of changes in mRNA expression were determined by calculating 2^−Δ Δ*C*t^.

### Immunohistochemistry staining and quantification of the results

Lens anterior capsule membranes were washed in 0.01 M phosphate-buffered saline (PBS) and attached to a slide with the lens epithelial cells facing upward. Capsule preparations were fixed in acetone for 10 min at 4°C, incubated with 3% hydrogen peroxide for 10 min to quench endogenous peroxidase activity, and then blocked with 10% normal goat serum for 20 min at room temperature. The capsules were then incubated with mouse monoclonal anti-human αA-crystallin (dilution 1∶150; Abcam, Inc., Cambridge, MA, USA) overnight at 4°C, rinsed with PBS, and incubated with horseradish peroxidase-conjugated secondary antibody (dilution 1:200; Abcam, Inc.) for 30 min at room temperature. The slides were stained with 3,3′-diaminobenzidine tetrahydrochloride (Beyotime, Jiangsu, China) and counterstained with hematoxylin. Finally, the slides were washed consecutively in 80%, 95%, and 100% alcohol, and xylene, and then sealed with nail polish. The lens anterior capsule membranes were then observed under a light microscope and photographed.

Immunohistochemistry (IHC) images were analyzed using Image-Pro® Plus 6.0 software (Media Cybernetics, Inc., Bethesda, MD, USA) as previously described [20]–[22]. Anterior capsule membrane samples from three patients per experimental group were used in each experiment. Tissue staining in each sample was measured in at least six different fields. The total integrated optical density (IOD) of the area of interest (AOI) in each field was recorded. Data are presented as the mean density of the immunoreactive area (IOD/AOI).

### Statistical analysis

Data are expressed as means ± standard deviation. Logistic regression was used to analyze the association between high myopia and dark nuclear cataract. Continuous parameters were compared by one-way analysis of variance followed by the least significant difference test. P-values < 0.05 were considered statistically significant. All analyses were performed using SPSS software (version 11.0; SPSS Inc., Chicago, IL, USA).

## Results

### High myopia is a risk factor for dark nuclear cataract

**Figure 1:**
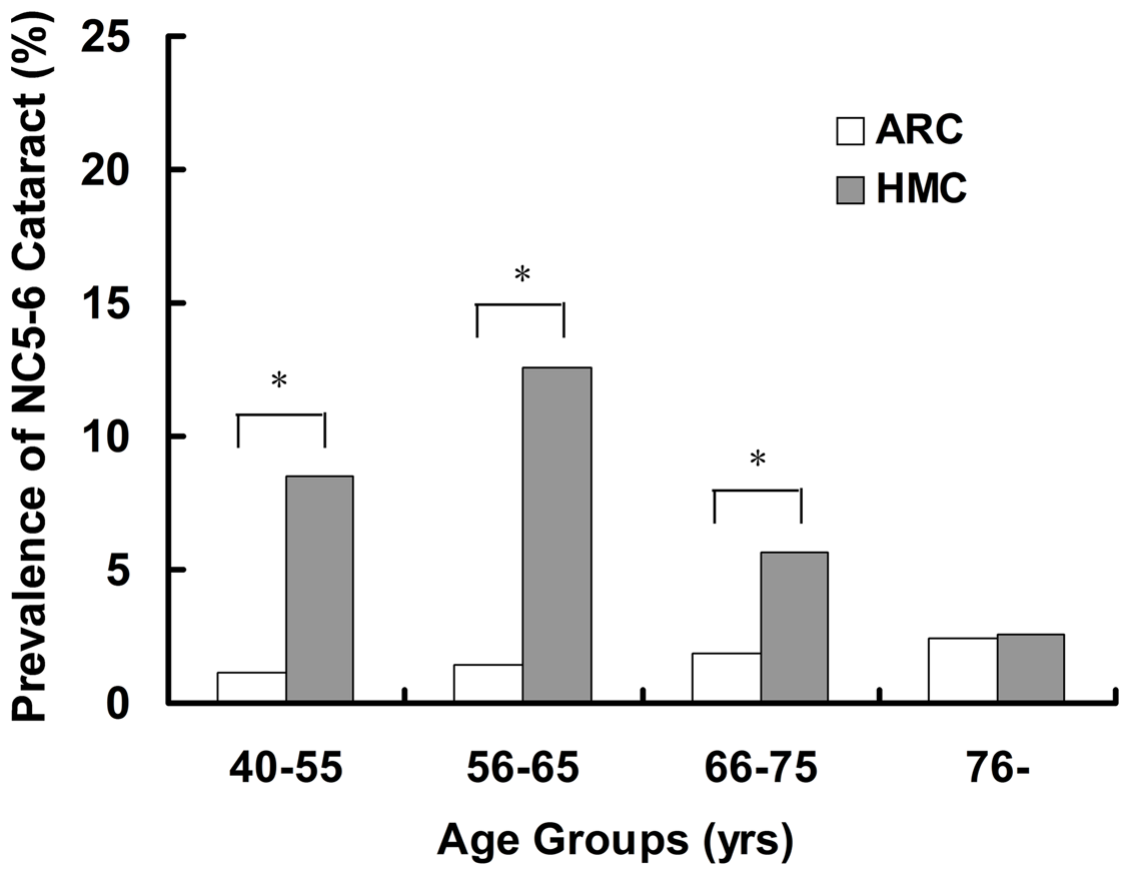
Prevalence of Grade Nuclear Color 5–6 cataracts in different age groups among age-related cataract and high-myopic cataract. Statistically significant differences were evident in the 40–55, 56–65, and 66–75 years age groups (Pearson’s chi-squared test; *all p<0.05).

Table 1 shows the number of HMC and ARC patients who presented with dark nuclear cataract (NC5–6) and underwent cataract surgery at our center in 2012. High myopia was associated with a significantly greater risk of dark nuclear cataract (odds ratio [OR]: 5.16; 95% confidence interval [CI]: 3.98–6.69; p<0.001). The mean patient ages in the ARC NC2–4, HMC NC2–4, ARC NC5–6, and HMC NC5–6 Groups were 69.9±16.4, 68.7±13.8, 71.4±11.6, and 59.6±8.3 years, respectively. Of all four groups, the mean age at cataract onset was significantly lower in the HMC NC5–6 Group (all p<0.05). Figure 1 shows the prevalence of NC5–6 cataract in different age groups of ARC and HMC patients. Statistically significant differences were evident for the 40–55, 56–65, and 66–75 years age groups (all p<0.05). The male: female ratio was 1124:1057 in the HMC NC2–4 Group and 108:90 in the HMC NC5–6 Group. Gender was not associated with the prevalence of NC5–6 cataract in HMC patients (p > 0.05).

**Table 1:** Number of patients with a dark nucleus (Nuclear Color 5–6) in each group.

Category	§NC5-6	NC2-4
† ARC	85	4833
‡HMC	198	2181

† age-related cataract.

‡ high-myopic cataract.

§ nuclear color.

### General characteristics of the samples

The general characteristics of patients in each group are summarized in Table 2. The mean age of patients in the HMC NC5–6 Group was lower than that of patients in the ARC NC2–3, HMC NC2–3, and ARC NC5–6 Groups. There were also more men in the HMC NC5–6 Group than in the other four groups (all p *>* 0.05).

**Table 2:** Baseline characteristics of the samples.

Category	Control	†ARC §NC2-3	‡HMC NC2-3	ARC NC5-6	HMC NC5-6
Eyes (n)	9	7	7	8	11
Sex male	4	3	3	4	7
female	5	4	4	4	4
Age (yrs)	35.8±3.5	66.3±2.7	67.7±4.3	70.7±3.4	60.3±4.1
Axial length (mm)	23.12±0.78	23.69±0.61	29.74±1.68	23.45±0.55	30.69±1.75

† age-related cataract.

‡ high-myopic cataract.

§ nuclear color.

### CpG island methylation in the *αA-crystallin* promoter was similar in age-related cataract and high-myopic cataract classified as Lens Opacity Classification System III Grade Nuclear Color 2–3

**Figure 2:**
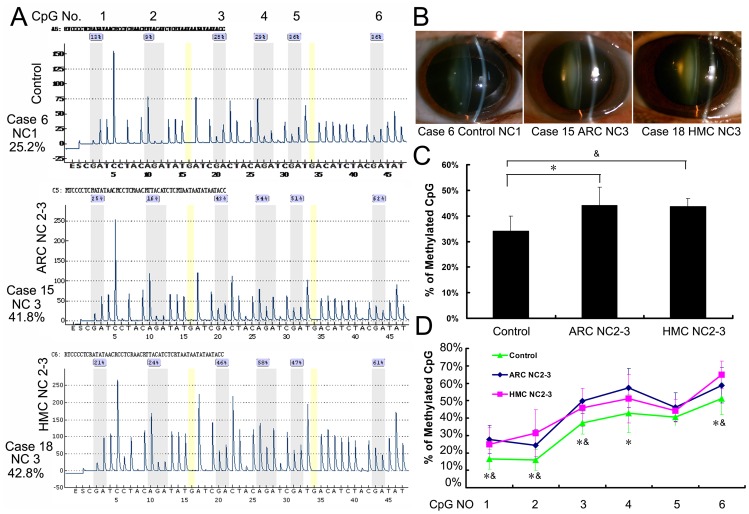
CpG island methylation in the *αA-crystallin* promoter was similar in age-related cataracts and high-myopic cataracts classified as Lens Opacity Classification System III Grade Nuclear Color 2–3. (A) Representative Pyrosequencing results of the Control, Age-Related Cataract (ARC) Nuclear Color (NC) 2–3 and High-Myopic Cataract (HMC) NC2–3 Groups. (B) Anterior segment photos of patients in each group. (C) The six selected CpG islands in the ARC NC2–3 (44±8.3%) and HMC NC2–3 (43.7±3.8%) Groups displayed higher methylation compared with the Control Group (34±6.1%). (D) Percentage of methylation at each individual CpG site in each group. (*Statistically significant differences were found between the Control and ARC NC2–3 Groups; ^&^statistically significant differences were found between the Control and HMC NC2–3 Groups; *^,&^all p<0.05. However, no statistically significant differences were found between the two cataract groups.)

Pyrosequencing, a real-time DNA sequencing method, was used to determine single-base variations caused by CpG methylation of the *CRYAA* promoter in each group. Figure 2 shows the methylation status of the *CRYAA* promoter in the Control, ARC NC2–3, and HMC NC2–3 Groups. Methylation of the six selected CpG islands was significantly greater in the ARC NC2–3 (44±8.3%) and HMC NC2–3 (43.7±3.8%) Groups than in the Control Group (34±6.1%; both p<0.05). However, methylation of the *CYRAA* promoter was not significantly different between the ARC NC2–3 and HMC NC2–3 Groups.

### Expression of αA-crystallin was similar in age-related cataract and high-myopic cataract classified as Lens Opacity Classification System III Grade Nuclear Color 2–3

**Figure 3:**
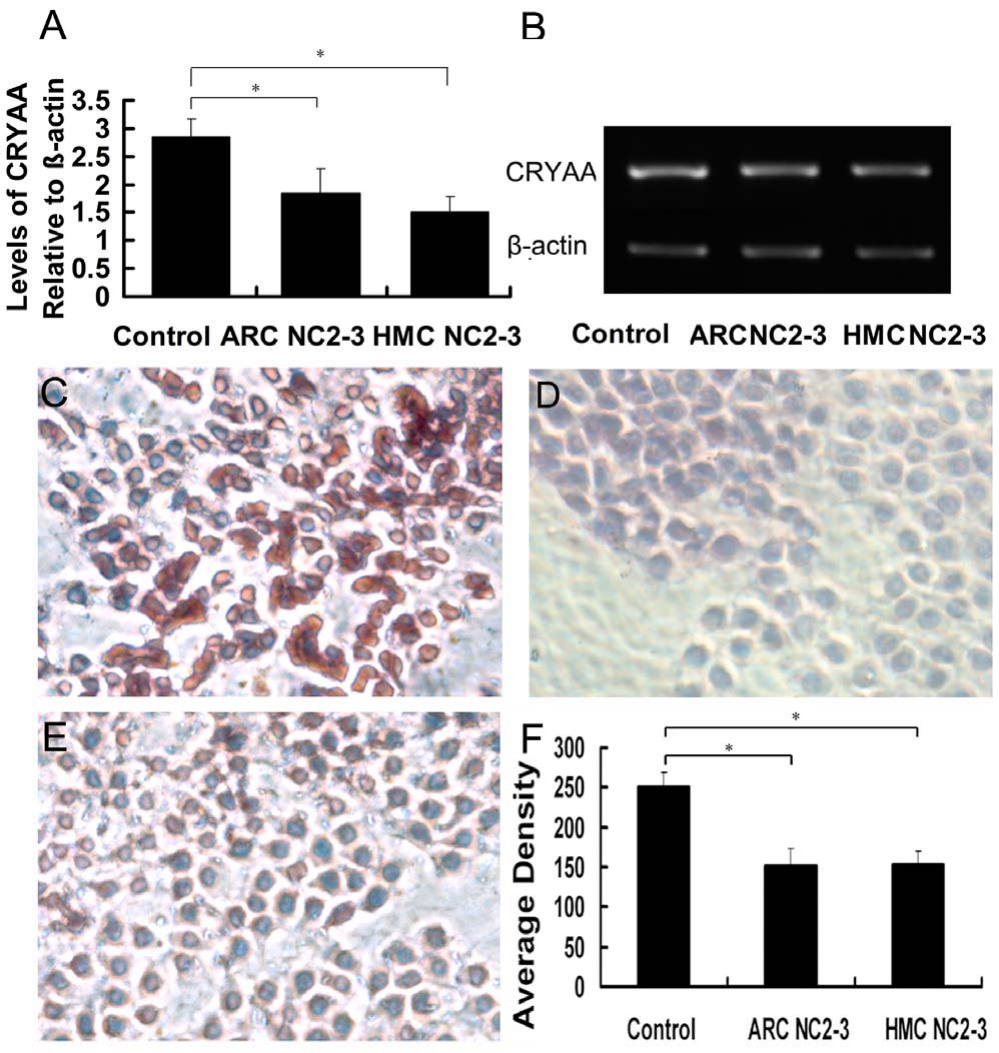
Expression of *αA-crystallin* was similar in age-related cataracts and high-myopic cataracts classified as Lens Opacity Classification System III Grade Nuclear Color 2–3. Alpha-crystallin expression in the lens epithelium of each group was detected by (A) real-time polymerase chain reaction (PCR; also showing levels of *αA-crystallin* relative to *β-actin*) and (B) reverse transcription PCR (C, D, E) Representative immunohistochemistry images of the Control, Age-Related Cataract Nuclear Color (NC) 2–3, and High-Myopic Cataract NC2–3 Groups. (F) Mean staining density in each group (*all p<0.05).

To determine the expression of αA-crystallin, we conducted real-time PCR, RT-PCR, and IHC using lens anterior capsule tissue samples from each group. Real-time PCR and RT-PCR (Figure 3A and B) showed that *CRYAA* mRNA expression in the ARC NC2–3 and HMC NC2–3 Groups was 64.8% and 52.8% of that of Control Group, respectively (both p<0.05). No statistically significant differences were found between the two cataract groups. Likewise, Figure 3C–F shows that αA-crystallin expression (mean density) was significantly lower in the ARC NC2–3 (151.5±22.3) and HMC NC2–3 (155.9±17.4) Groups than the Control Group (249.9±19.2; both p<0.05). However, αA-crystallin expression was not significantly different between the ARC NC2–3 and HMC NC2–3 Groups.

### CpG island methylation in the *αA-crystallin* promoter was significantly greater in high-myopic cataract than in age-related cataract classified as Lens Opacity Classification System III Grade Nuclear Color 5–6

**Figure 4:**
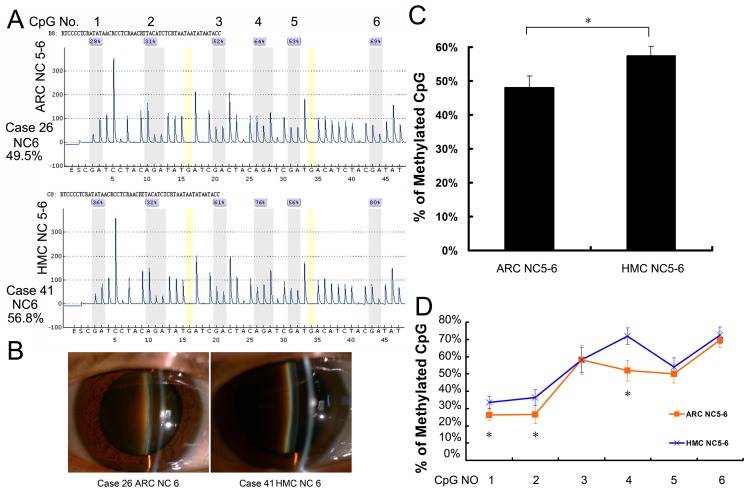
CpG island methylation in the *αA-crystallin* promoter was significantly greater in high-myopic cataracts than in age-related cataracts classified as Lens Opacity Classification System III Grade Nuclear Color 5–6. (A) Representative pyrosequencing results of the Age-Related Cataract (ARC) Nuclear Color (NC) 5–6 and High-Myopic Cataract (HMC) NC5–6 Groups. (B) Anterior segment photos of patients in each group. (C) The six selected CpG islands in the HMC NC5–6 Group (57.6±2.6%) displayed hypermethylation compared with the ARC NC5–6 Group (48.1±3.4%). (D) Percentage of methylation at each individual CpG site in each group. (*Statistically significant differences were found between the ARC NC5–6 and HMC NC5–6 Groups; *all p<0.05).

Figure 4 shows that, among patients classified as LOCS III Grade NC5–6, CpG island methylation in the *CRYAA* promoter was significantly greater in the HMC Group (57.6±2.6%) than in the ARC Group (48.1±3.4%; p  = 0.01), even though mean patient age in the HMC group was lower (Tables 1: p<0.05 and Table 2: p *>* 0.05). Methylation was also significantly greater in the HMC NC5–6 Group than in the axial length-matched cataract Control Group (i.e. HMC NC2–3; p<0.001). In fact, it was higher in the HMC NC5–6 Group than in all other groups (all p<0.05). Therefore, in patients with high myopia, dark nuclear cataract develops at an earlier age and the lens epithelium exhibits greater methylation of the *CRYAA* promoter compared with axial length-matched or age-matched cataract controls.

### AlphaΑ-crystallin expression in the lens epithelium was significantly lower in the HMC NC5–6 Group than in the ARC NC5–6 Group

**Figure 5:**
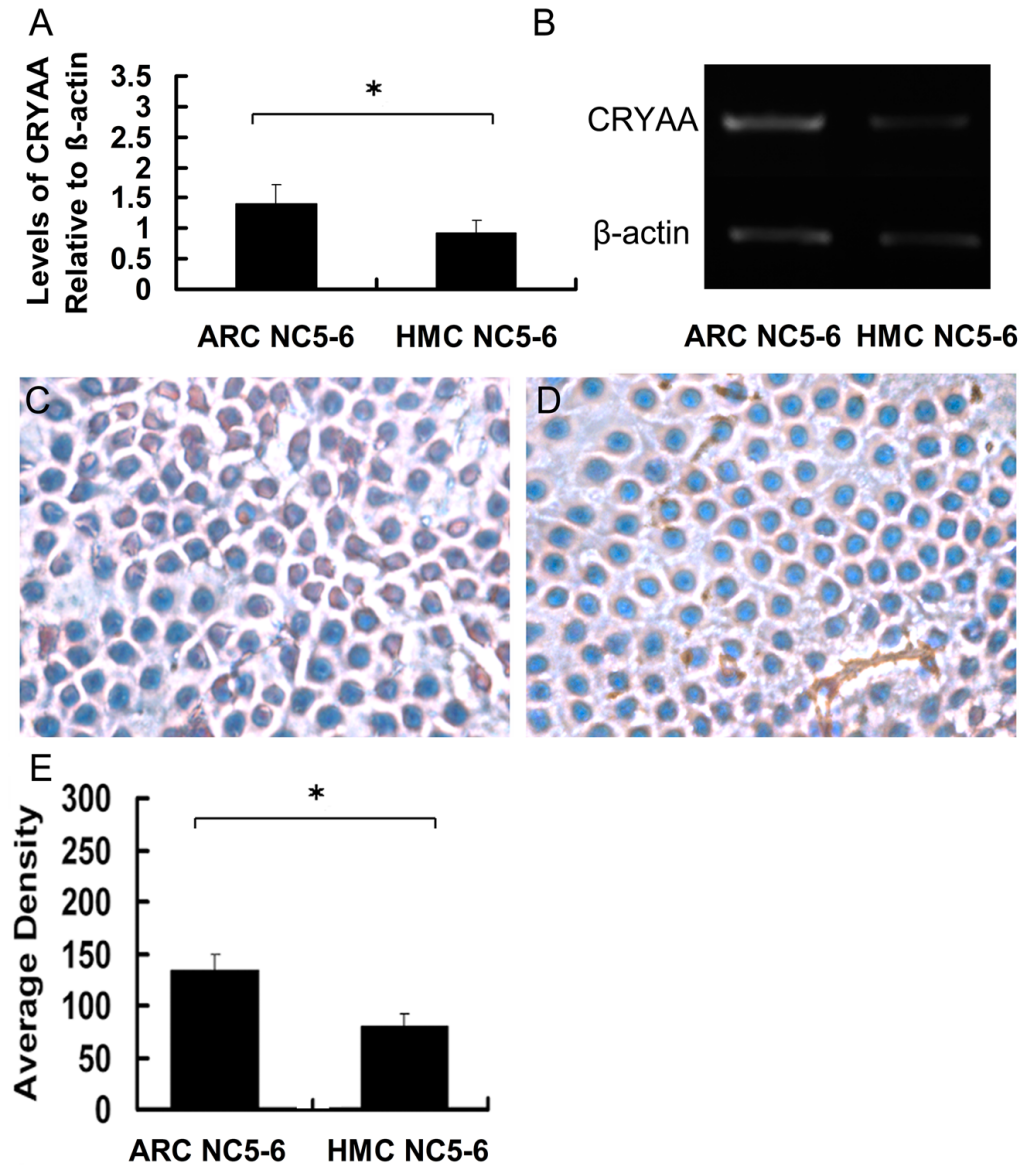
αΑ-crystallin expression in the lens epithelium was significantly lower in the High-Myopic Cataract Nuclear Color 5–6 Group than in the Age-Related Cataract Nuclear Color 5–6 Group. αA-crystallin expression in the lens epithelium of each group was detected by (A) real-time polymerase chain reaction (PCR; also showing levels of *αA-crystallin* relative to *β-actin*) and (B) reverse transcription PCR. (C, D) Representative immunohistochemistry images of the Age-Related Cataract Nuclear Color (NC) 5–6 and High-Myopic Cataract NC5–6 Groups. (E) Mean staining density in each group (*both p<0.05).

We next performed real-time PCR, RT-PCR, and IHC using lens epithelial cell samples from the ARC NC5–6 and HMC NC5–6 Groups. Real-time PCR and RT-PCR (Figure 5A and B) showed that *CRYAA* mRNA expression in the HMC NC5–6 Group was 32.2% of that of Control Group and 65.7% of that of the ARC NC5–6 Group (both p<0.05). Meanwhile, IHC results showed that αA-crystallin expression (mean density) in the lens epithelium was significantly lower in the HMC NC5–6 Group (79.8±12.6) than in the ARC NC5–6 Group (133.6±15; Figure 5C–E). Likewise, of all five experimental groups, αA-crystallin expression was lowest in the HMC NC5–6 Group (all p<0.05).

## Discussion

High myopia is a common eye disease in Asian populations. It is closely associated with many pathologic changes of the eye, including marked fundus changes, open-angled glaucoma [23], [24], and the early onset of cataract [25], [26]. However, in the last 50 years, few studies have tried to elucidate the etiology of HMC, especially of dark nuclear cataract. In this study, we found that high myopia is a risk factor for dark nuclear cataract, and that CpG islands in the *CRYAA* promoter are hypermethylated in the lens epithelial cells of patients with high-myopic dark nuclear cataract compared with patients with ARC of the same LOCS III grade. Real-time PCR, RT-PCR, and IHC confirmed that αA-crystallin expression is also significantly downregulated in patients with high-myopic dark nuclear cataract than in patients with ARC of the same LOCS III grade. Overall, our findings suggest that downregulation of *CRYAA* associated with DNA hypermethylation is involved in the development of high-myopic dark nuclear cataract.

Increasing clinical evidence suggests that high myopia is a risk factor for dark nuclear cataract. For example, epidemiologic studies conducted in India [2], [7] revealed that mean cataract density was higher in patients with high myopia. However, they did not report whether high myopia was associated with dark nuclear cataract. At our clinic, we frequently encounter patients with high myopia, who often present with brown or dark nuclear cataract at a relatively young age. Thus, we believed the incidence of dark nuclear cataract to be higher in patients with high myopia than in patients with ARC. Therefore, we reviewed the clinical data of patients who underwent cataract surgery at our clinic in 2012, and found that high myopia was associated with a significantly greater risk of dark nuclear cataract (OR: 5.16; 95% CI: 3.98–6.69; p<0.001).

Increased oxygen tension caused by earlier vitreous liquefaction in patients with high myopia relative to patients with ARC could be an important cause of the earlier onset of dark nuclear cataract in HMC patients, according to studies of cataract formation after vitrectomy [27], [28]. Of note, increased oxygen tension around the lens exposes lens proteins to marked oxidative stress in high-myopic patients [28], [29]. Additionally, studies comparing the oxidative consumption of glutathione in the lenses of HMC and ARC patients [30], [31] showed that, compared with healthy controls, the lenses of patients with cataract had lower glutathione levels, with the lowest levels found in the lenses of high-myopic patients. Also, elevated malondialdehyde levels were found in the cataractous lenses and vitreous of myopic patients compared with nonmyopic patients with cataract [30]. Therefore, the lens needs to maintain a healthy defense system to protect against oxidative stress, particularly in patients with high myopia.

Alpha-crystallin, the best-characterized structural protein of the human lens, reportedly acts as a chaperone under conditions of oxidative stress, thereby maintaining the transparency of the lens [10], [32]. However, in this study, we observed a significant decrease in αA-crystallin expression in patients with high-myopic dark nuclear cataract compared with patients with ARC of similar severity. This reduction in αA-crystallin expression may increase the susceptibility of lens proteins to oxidative stress associated with aging.

Epigenetic regulation of gene expression was recently shown to be associated with many eye diseases, including age-related macular degeneration [33], glaucoma [34], and retinoblastoma [35]. Additionally, we reported [14] that hypermethylation of CpG islands in the *CRYAA* promoter downregulates αA-crystallin expression in ARC. The role of hypermethylation in the downregulation of *CRYAA* expression was confirmed by treating lens epithelial cells with a demethylating agent, which restored *CRYAA* expression [14]. Therefore, we wondered whether the epigenetic regulation of *CRYAA* expression also functioned in the pathogenesis of HMC. Our analysis showed that hypermethylation of CpG islands in the *CRYAA* promoter was much greater in patients with high-myopic dark nuclear cataract than in patients with ARC of a similar LOCS III grade. Consistent with this, we found that much less αA-crystallin was synthesized in the lenses of patients with high-myopic dark nuclear cataract.

**Figure 6:**
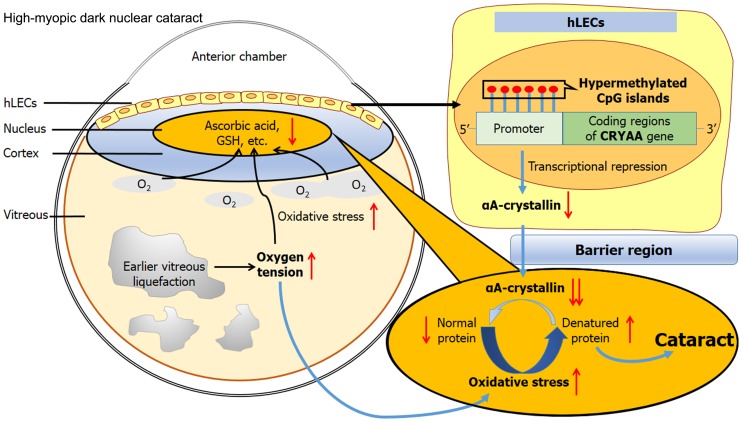
Illustration for the possible mechanism of high-myopia-induced dark nuclear cataract.

It is established that fewer reducing agents, such as glutathione [36] and ascorbic acid, are present in the nuclear region of the lens than in the cortical region, leading to a localized reduction in antioxidative capacity. Consequently, a greater proportion of proteins are denatured as a result of oxidative stress in this region. In the lenses of the young, a sufficient number of chaperones (the majority αA-crystallin) are expressed to bind these denatured proteins, preserving the transparency of the lens. However, in high-myopic patients, much less αA-crystallin is synthesized to pass into the nucleus and restore these denatured proteins. This may accelerate the oxidative modification of proteins in the nucleus, resulting in the development of high-myopic dark nuclear cataract. Figure 6 summarizes the pathogenesis of high-myopia-induced dark nuclear cataract.

While collecting lens anterior capsule membranes over a period of 4 months, it became apparent that patients with high-myopic dark nuclear cataract were younger than other groups of patients. These patients visited our clinic at an earlier age because of a rapid deterioration in their visual acuity. The significantly higher prevalence of Grade NC5–6 cataract in the younger age groups of HMC patients further indicated that dark nuclear cataract occurs at an earlier age in patients with HMC than in patients with ARC. In contrast, patients with HMC without a dark nucleus were admitted for cataract surgery at up to 80 years of age, often with cataract classified as LOCS III Grade NC2. Our clinical experience is also supported by our current data, which show that methylation of CpG islands in the *CRYAA* promoter is significantly lower in high-myopic patients with soft nuclear cataract (NC2–3) compared with patients with dark nuclear cataract, but similar to that in ARC classified as NC2–3. Therefore, we believe that two types of HMC may exist. The first is characterized by the early onset and rapid progression of nuclear cataract, and typically presents with a dark nucleus. The second is characterized by gradual progression, and may retain a soft nucleus classified as NC2–3, even in older patients. Further studies are needed to elucidate the causes of this phenomenon. On the other hand, although the proportion of men was higher in the samples we collected, gender was not associated with the prevalence of dark nucleus in this study. Larger, population-based studies are still warranted in future.

In conclusion, our data indicate that high myopia is a risk factor for dark nuclear cataract, and that hypermethylation of CpG islands in the *CRYAA* promoter leading to downregulation of αA-crystallin, a major component of antioxidative mechanisms within the lens, functions in the pathogenesis of high-myopic dark nuclear cataract. These results provide a new direction for research into HMC.
